# Tumor-Associated Macrophages as Key Modulators of Disease Progression in Diffuse Large B-Cell Lymphoma

**DOI:** 10.3390/biomedicines13051099

**Published:** 2025-05-01

**Authors:** Corina Joldes, Laura Jimbu, Oana Mesaros, Mihnea Zdrenghea, Bogdan Fetica

**Affiliations:** 1 Department of Hematology, Iuliu Hatieganu University of Medicine and Pharmacy, 8 Babes Street, 400012 Cluj-Napoca, Romania; ioana.jimbu@umfcluj.ro (L.J.); mesaros.oana@umfcluj.ro (O.M.); mzdrenghea@umfcluj.ro (M.Z.); 2 Department of Hematology, Ion Chiricuta Oncology Institute, 34–36 Republicii Street, 400015 Cluj-Napoca, Romania; 3 Department of Pathology, Ion Chiricuta Oncology Institute, 34–36 Republicii Street, 400015 Cluj-Napoca, Romania; feticab@yahoo.com

**Keywords:** macrophage polarization, TAMs, non-Hodgkin lymphoma

## Abstract

With the advent of new therapeutic approaches, there is hope that anticancer treatment will eventually be possible without the use of chemotherapy. Efficient immunotherapeutic options have recently emerged in many cancers, offering a less aggressive approach, with overall better tolerance, making them also suitable for frail patients. Response to immunotherapy relies on the availability, functionality, and efficacy of the host’s immune effector mechanisms. One of the key factors determining the efficacy of immunotherapy is the tumor microenvironment, which encompasses various immune effectors, including macrophages, which play a crucial role in regulating immune responses through phagocytosis and antigen presentation. Macrophages are prototypically divided, according to their polarization, into either the pro-inflammatory M1 type or the anti-inflammatory M2 type. In the tumor microenvironment, M2-polarized macrophages, known as tumor-associated macrophages (TAMs), are the predominant phenotype and are associated with tumor progression. The M1/M2 paradigm contributes to the understanding of tumor progression. Due to the variable microenvironment, the mechanisms regulating TAMs can vary across different cancers. Variations in TAM polarization may account for the different treatment responses in patients with similar diseases. This paper investigates the connection between TAMs, disease progression, and treatment responses in the most frequent solid hematologic cancer, diffuse large B-cell lymphoma.

## 1. Introduction

Lymphoma is a type of cancer characterized by the abnormal clonal proliferation of lymphoid tissue [1]. The term “lymphoma” encompasses a wide range of lymphoid malignancies, reflected in the complexity of classification systems. Historically, lymphoma has been divided into Hodgkin’s lymphoma (HL) and non-Hodgkin lymphoma (NHL) [2]. Since Thomas Hodgkin provided the first morphological description of this disease in 1832, the classification has evolved significantly, taking into account histological features, surface molecule expression on tumor cells, and molecular biology [3]. In 2022, the World Health Organization (WHO) released the fifth edition of the classification for lymphoid neoplasms, which divides these disorders into three categories: B-cell lymphoid proliferations and lymphomas, T-cell and NK-cell lymphoid proliferations and lymphomas, and stroma-derived neoplasms of lymphoid tissues [4]. The first group includes tumor-like lesions dominated by B-cells that were not classified previously, precursor B-cell neoplasms, and mature B-cell neoplasms. Mature B-cell neoplasms are further categorized into several subtypes: pre-neoplastic and neoplastic small lymphocytic proliferations, splenic B-cell lymphomas and leukemias, lymphoplasmacytic lymphoma, marginal zone lymphoma, follicular lymphoma, cutaneous follicle center lymphoma, mantle cell lymphoma, transformations of indolent B-cell lymphomas, large B-cell lymphomas, Burkitt lymphoma, and KSHV/HHV8-associated B-cell lymphoid proliferations and lymphomas. This group also includes lymphoid proliferations and lymphomas associated with immune deficiency and dysregulation, as well as Hodgkin lymphoma. The second category covers tumor-like lesions that are predominantly T-cell in nature, precursor T-cell neoplasms, and mature T-cell and NK-cell neoplasms. The third category includes mesenchymal dendritic cell neoplasms, myofibroblastic tumors, and spleen-specific vascular-stromal tumors, which were not included in previous classifications [4].

Recent data indicate that various lymphoid neoplasms, including diffuse large B-cell lymphoma (DLBCL), comprise distinct entities characterized by unique epidemiological and etiological profiles. Lymphoma is recognized as the sixth most common cancer worldwide [5]. In both the United States and Europe, the incidence of non-Hodgkin lymphoma (NHL) is approximately 20 cases per 100,000 individuals, with a higher prevalence observed in men [6]. NHL is identified as the most common form of hematological cancer across the world [7]. Notably, risk factors such as immunosuppression linked to HIV infection and the use of immunosuppressive medications can significantly elevate the risk of developing NHL [8]. The Epstein–Barr virus (EBV) has also been acknowledged as a contributing factor [9].

After the challenges of a correct lymphoma diagnosis, another essential goal is the selection of the right therapeutic approach. Unlike indolent lymphomas, where many patients may not need immediate treatment, and a ‘watch and wait’ approach is often used [10], aggressive lymphomas require a treatment plan focused on eradicating the disease [11]. The prototype for aggressive lymphoma is DLBCL, alongside the closely related B-cell malignancies like high-grade B-cell lymphoma (HGBL), gray zone lymphoma, Burkitt lymphoma (BL), and primary mediastinal large B-cell lymphoma (PMBL), collectively accounting for up to one-third of all lymphomas [12]. The CHOP chemotherapy regimen has long been the standard treatment for DLBCL, and adding the anti-CD20 monoclonal antibody rituximab has significantly improved patient survival rates [13]. We now have access to several innovative therapies, including monoclonal antibodies (mAbs), engineered immune effector proteins specifically targeting certain antigens [14]. There are also antibody-drug conjugates (ADCs), which consist of a monoclonal antibody linked to a drug that can enter and destroy targeted cells [15]. Additionally, chimeric antigen receptor (CAR) T-cell therapy utilizes patient T cells modified in the laboratory to attack cancer cells [16] effectively. Lastly, there is allogeneic stem cell transplantation (SCT), which involves the transfer of stem cells from a donor to a patient [16] and relies on the graft versus tumor effect for efficacy Treatment failures affect approximately 20% to 40% of cases, notably among patients facing relapse or resistance [17]. Therefore, it is imperative to investigate novel therapeutic targets to enhance treatment efficacy.

One of the paramount immune mechanisms is inflammation, and this process must be carefully regulated to prevent damage [18]. The inflammatory response has different phases, and macrophages are essential during the process initialization and repair phase [19]. It is worth noticing that macrophages can sometimes play a role in promoting the growth of cancer cells [20]. This occurs due to the ability of macrophages to reduce the immune response against cancer cells and inhibit apoptosis, or programmed cell death, which is a natural process to eliminate abnormal or damaged cells [21]. Including stromal cells, endothelial cells, and fibroblasts, the tumor microenvironment (TME) is also inhabited by cells belonging to the innate immune system: macrophages, dendritic cells, neutrophils, natural killer (N.K.) cells, and the adaptive immune system (T- and B-cells) [22]. Among these, macrophages are essential as they regulate the immune response through their ability to present antigens and phagocytose foreign substances [23]. Based on their activation stimuli, macrophages can be polarized towards the M1 pro-inflammatory type, or the M2 anti-inflammation type [23]. M1-like macrophages can eliminate pathogens and tumor cells by secreting pro-inflammatory cytokines, including interleukin (IL)-12p40 and tumor necrosis factor-alpha (TNF-α) [17]. In contrast, M2-like macrophages facilitate immunosuppression and immune escape during tumor progression by secreting anti-inflammatory cytokines, such as interleukin-10 (IL-10) and transforming growth factor-β (TGF-β). Additionally, they display a high level of expression of the mannose receptor (CD206) [17]. In the TME, M2-polarized macrophages are the prevalent type. They are referred to as tumor-associated macrophages (TAMs), involved in cancer development [24], infiltration, dissemination, and immune escape, particularly in the context of DLBCL [17]. These factors strongly associate with unfavorable outcomes in various malignancies, including lymphomas [21].

## 2. Macrophage Polarization

**Figure 1:**
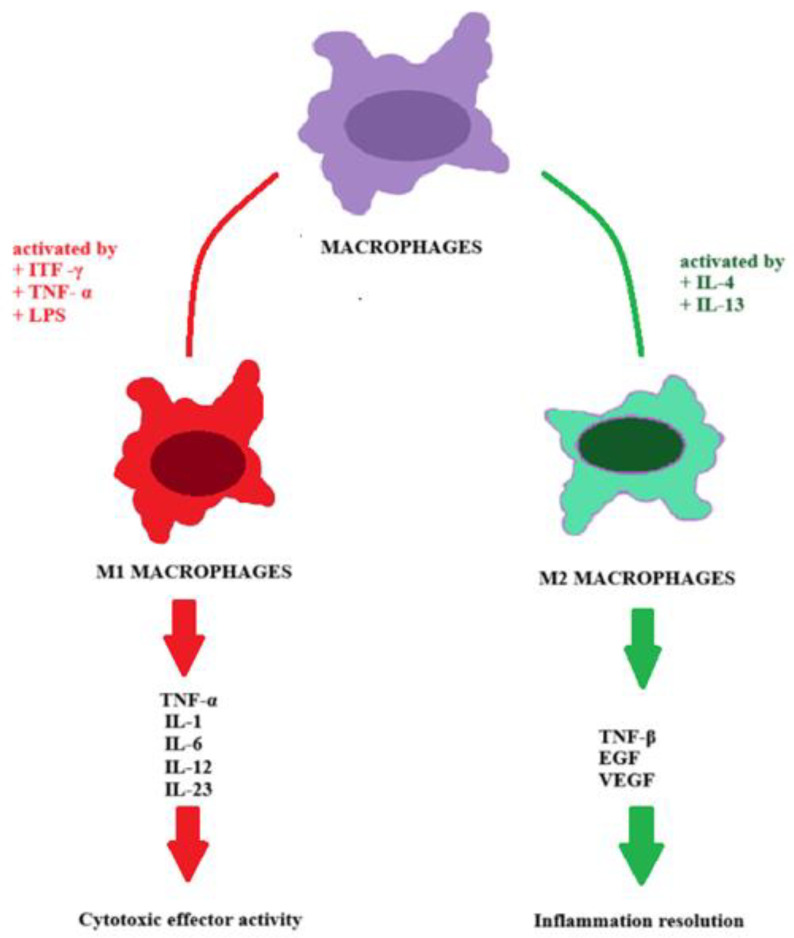
Macrophage Polarization. Activation of macrophages by IFN-y, TNF-a, or LPS induces the M1 phenotype, which releases pro-inflammatory cytokines with cytotoxic and anti-tumor effects. Conversely, activation by IL-4 and IL-13 leads to the M2 subtype, which secretes cytokines such as TGF-ß and VEGF, associated with anti-inflammatory responses and tumor progression.

In the 1990s, macrophages were classified as either M1 classically activated or M2 alternatively activated, each with unique functional roles based on phenotype [18]. Two distinct categories of tissue-resident macrophages are categorized based on their location—blood monocyte-derived macrophages (MDMs) and tissue-resident macrophages [25]. Blood monocyte evolution happens in the bone marrow, and after they attain maturity, they pass into the bloodstream, where they can stay for several days. After receiving different stimuli from distinct cytokines and chemokines, they migrate to diverse tissues and differentiate into macrophages. There, they can be involved in restoring the tissue macrophage pool or experiencing apoptosis [26]. On the other hand, a part of the tissue-resident macrophages evolve from the yolk sac individually from the hematopoietic stem cells and migrate first to the fetal liver and then to various tissues [27]. In typical circumstances, the regional expansion of tissue-resident macrophages is restricted. In inflammation or depletion of macrophages, there is a significant surge in their expansion within the region [28]. Macrophages are diverse and flexible cells found in many organs [25]. In response to various stimuli, such as inflammation or infection, macrophages can modify their phenotype during activation [18], leading to their polarization, reflecting the influence of different stimuli on macrophage development [29]. The paradigm of M1 vs. M2 closely resembles the T-helper 1 (Th1) and T-helper 2 (Th2) division [30]. Macrophages differentiate into the M1 subtype when activated by Th1 cytokines, such as interferon-γ (IFN-γ) and tumor necrosis factor-α (TNF-α), as well as by pathogen-associated components like lipopolysaccharide (LPS) [18]. After activation, macrophages release cytotoxic and anti-proliferative effects by producing pro-inflammatory cytokines, including TNF-α, IL-1, IL-6, IL-12, and IL-23 [18]. IL-4 and IL-13 effectively activate the alternative pathway, thereby promoting the activation of the M2 subtype of macrophages [30]. This phenotype plays a crucial role in resolving inflammation through the actions of transforming growth factor-β (TGF-β), epidermal growth factor (EGF), and vascular endothelial growth factor (VEGF) (Figure 1) [23]. According to a specific interpretation, M1 macrophages are formed when triggered by the growth factor granulocyte-macrophage colony-stimulating factor (GM-CSF). In contrast, the M2 subtype develops following activation with macrophage colony-stimulating factor (M-CSF). However, there is insufficient information to establish a clear correlation between the activation by GM-CSF/M-CSF and the polarization of M1/M2 macrophages [31]. Numerous studies on macrophages indicate that their presence in the TME may be influenced by chemokine ligands produced by cancer cells [32]. Various types of chemotactic cytokines have been identified and classified into four categories: CXC, CC, C, and CX3C [33]. Initially, TAMs were believed to fight cancer [34], but studies have shown they promote tumor progression by lowering immune response and apoptosis while promoting angiogenesis and genetic instability [21]. First, TAMs are enlisted and, afterward, polarized into a type that can promote tumor growth and survival [35,36,37]. The process is initiated by various substances, including CCL2, CCL5, CCL7, CXCL1, and vascular endothelial growth factor (VEGF) [36]. Macrophage polarization is a complex topic, and the traditional classification into M1 and M2 subtypes often overlooks their plasticity [38]. To address this limitation, M2 macrophages have been further divided into four subsets, M2a, M2b, M2c, and M2d, based on their phenotype [39], with each activated by specific factors [40]. Recently, a new subtype of macrophages known as mixed-polarized macrophages has been described [38]. The distribution and polarization status of these macrophages have been investigated in various tumor regions, including tumor tissues and stromal areas, to gain insights into their functions [38]. The presence of mixed phenotype macrophages in pulmonary cancer has been observed to be intricately linked to the infiltration of tumor sites by tissue-resident memory T-cells and the promising potential of immunotherapy [41]. In the context of gastric cancer that presents with diffuse cells, there is ongoing inquiry into whether mixed-phenotype macrophages serve as a key component within the TME, as IL-1β derived from this subtype activates fibroblasts and collaboratively creates a tumor-promoting microenvironment [38]. Their potential role in influencing tumor progression, including hematologic malignancies, warrants further investigation. To develop more effective therapies for hematologic malignancies, it is crucial to conduct further research on TAMs. These cells are closely linked to tumor growth, and understanding their role can provide valuable treatment targets.

## 3. What Is the Role of TAMs in DLBCL?

Currently, six types of TAMs are recognized: immunosuppressive, angiogenetic, metastasis-associated, invasive, activated, and perivascular [21]. As mentioned, depending on their polarization, TAMs can demonstrate either antitumor or pro-tumorigenic effects. The connection between TAMs, tumor evolution, and outcome has been established for various hematological malignancies [21]. For DLBCL, it is thought that TAM polarization can be influenced by apoptotic NHL cells, which exhibit reduced galectin-3 expression for this subtype. [18]. It was described that, in vitro, macrophages can help NHL cells grow when they come into contact. It is thought that TAMs release signals like C5a, IL-6, and TNF-α. These signals then activate the STAT3 and NF-kB pathways [42]. Also, TAMs are involved in extracellular matrix remodeling via legumain, leading to fibronectin degradation and angiogenesis [43]. A higher level of CD163e expression, a marker for the M2 phenotype, was associated with neovascularization found in the interfollicular area [44]. The identified oncogenic signaling pathways represent promising targets, based on extensive research into various lymphoma subgroups [45,46,47,48]. Recent observations suggest that the TME components of DLBCL are organized into composite cell neighborhood types (CNTs), each with distinct characteristics, spatial distributions, and functional roles, modulating tumor arrangement and immune cell presence [49]. The form of DLBCL associated with an immune deficit is characterized by reduced levels of immune cells and increased levels of tumor cells. It was noted that areas far from blood vessels lack immune cells, while regions near the vessels show only minimal immune cell infiltration. This pattern is observed across all genetic subtypes. [49]. The dendritic cell-enriched (DC-enriched) type features regions with fewer immune cells away from blood vessels, while closer areas show a higher concentration of cells marked by immune activity biomarkers, aspects seen in all genetic variants. Notably, there is a significant presence of CD11c+ DCs and antigen-experienced T cells, which are predominantly located near CD31+ blood vessels. This presentation is consistent with a heightened level of immune activity [49]. Cases with macrophage-enriched tumor immune microenvironments (Mac-enriched) often lack tumor cells and are rich in immune cells, particularly CD163+ macrophages and CD8 T cells. These environments show increased IDO-1 and LAG-3 expression, decreased HLA-DR, and genetic markers indicative of immune evasion [49].

In order to overcome chemotherapy resistance in cancer treatment, it is important to understand TAMs and their role in the effectiveness of current chemotherapy treatments, as they can create an immunosuppressive microenvironment that inhibits the immune system from effectively attacking tumor cells [50]. Secreting a variety of cytokines and growth factors, they can help tumor cells survive chemotherapy via survival pathways, like PI3K/AKT or NFkB pathways [51]. Also, by promoting the formation of new blood vessels, TAMs can decrease the effectiveness of treatment [52]. Targeting TAMs is an active area of research to improve chemotherapy efficacy and overcome resistance.

Based on the latest published data, we further review markers of poor prognosis in DLBCL and their association with macrophage polarization.

### 3.1. Galectin-3

During pre-clinical studies related to DLBCL, it has been observed that apoptotic NHL cells can encourage M2 polarization [21]. M2 has a decreased galectin-3 expression [18]. This glycoprotein is implicated in clearing apoptotic cells [53]. Galectins belong to the group of internal lectins and play a significant role in cell differentiation, progression, cell death, cell adherence, and migration [54]. Galectin-3 is prominently found in human tissues, playing a crucial role in a variety of immune cells, including macrophages, monocytes, dendritic cells, eosinophils, mast cells, natural killer cells, and activated T and B cells, along with endothelial cells and sensory neurons. Its widespread presence underscores its significance in immune function [55,56]. Galectin-3 has different functions based on its location: in the nucleus, it regulates pre-mRNA splicing and transcription; in the cytoplasm, it supports cell survival; and on the cell surface, it modulates cell interactions and epithelial cell–extracellular matrix interactions [57]. When studied as a potential biomarker for the prognosis of DLBCL, elevated levels of galectin-3 have been associated with more aggressive disease and poor prognosis, having a role in promoting tumor cell adhesion to the extracellular matrix, demonstrating anti-apoptotic properties, and being involved in differentiation from follicular lymphoma [58]. The molecule in question undergoes transport from the nucleus to the cytoplasm through non-classical secretory pathways, ultimately reaching the cell surface and beyond [58,59]. In the cytoplasm, it inhibits cell death by binding to ligands like Bcl-2, CD-95, and ALIX/AIP1 [60]. Galectin-3 is also involved in other pathologies. In renal cell carcinoma (RCC), galectin-3 expression rates are significantly elevated in the serum and tumor tissues of patients with renal cell carcinoma [61]. Emerging research has highlighted the remarkable potential of galectin-3 as a diagnostic tool for identifying malignant lesions in thyroid cancer [62]. In hepatocellular carcinoma (HCC) it facilitates the advancement of cancer progression [57]. In colorectal cancer, this method distinguishes patients with liver metastasis from those without. Higher levels detected during tumor removal are linked to lower survival rates over 10 years [63]. Upregulated expression was also observed in breast cancer, gastric cancer, and pancreatic carcinoma [57]. An experimental drug, GCS-100, a galectin-3 inhibitor, was investigated in clinical trials, including those for lymphoma. It aims to disrupt the TME and enhance the immune system activity. The potential of GCS-100 in the treatment of relapsed or refractory multiple myeloma and diffuse large B-cell lymphoma was observed, and phase 1/2 clinical trials for both indications were planned (NCT00609817; NCT00776802). However, these trials were ultimately discontinued due to insufficient funding [64].

### 3.2. CD68, CD163, and the CD163/CD68 Ratio

In vitro, studies have shown that macrophages can lead to NHL cell proliferation if in contact, being able to generate cytokines like C5a, IL-6, and TNF-α, activating STAT3 and NF-kB pathways [43]. Also, in DLBCL, M2 can alter the extracellular matrix, causing fibronectin deterioration and increasing angiogenesis [40]. Several clinical studies have investigated the role of TAMs in DLBCL prognosis, utilizing CD68, CD163, and the CD163/CD68 ratio as markers for M2 polarization [18]. A positive association between infiltration of TAM CD68-positive cells and improved survival in patients with DLBCL was demonstrated [18]. Studies have shown that the expression of CD68 is linked to a favorable outcome in patients who received a combination of rituximab and chemotherapy compared to those who only received chemotherapy [31]. An elevated CD163/CD68 ratio, evocative of M2 polarization, was observed in published data to anticipate poor outcomes [18]. It was indicated that patients with high levels of CD68 expression who received immunotherapy and chemotherapy had improved overall survival, while those with an elevated CD163/CD68 ratio experienced reduced progression-free survival and overall survival with the same treatment modality [32]. Both CD68 and CD163 are expressed in several pathologies beyond DLBCL. In patients with classical Hodgkin lymphoma (cHL), a comparison between cases with complete response (CR) and those without showed that the CR group had lower expressions of CD68 and CD163. This finding suggests that these markers are significant predictors of a complete response in cHL cases [65]. Additionally, for mycosis fungoides (MF) and Sézary syndrome (SS), the high ratio of CD163 to CD68 in the tumor stages of MF and SS indicates M2 polarization of tumor-associated macrophages (TAMs) and correlates with tumor progression [66].

### 3.3. PTX3

In a study, macrophage markers including CD68 (pan-macrophages), CD16 (M1-like), CD163 (M2-like), pentraxin3 (PTX3), and IL-10 were employed to examine the relationship between various TAM infiltrations and prognosis in DLBCL [67]. DLBCL is classified into two subtypes: germinal-center B-cell-like (GCB), with a better outcome, and activated-B-cell-like (ABC/non-GCB), with a more aggressive evolution [68]. The findings regarding the ABC/non-GCB subtype prognosis are somewhat associated with the presence of macrophages and the activation of the NF-kB pathway [67]. M2c macrophages regulate the immune system by utilizing markers such as CD163, PTX3, and IL-10 and by producing regulatory T lymphocytes (Tregs). PTX3, also known as TNF-inducible gene 14 protein (TSG-14), plays a role in regulating the inflammatory activity of macrophages. It is specifically expressed in macrophages exhibiting M2-like polarization, particularly the M2c-like subtype [30], but its role in lymphoid malignancies is still unclear. The markers linked with a bad prognosis were macrophages with high expression of CD16, CD68, microphthalmia-associated transcription factor (MITF), CD163, PTX3, and IL-10 and low infiltration of regulatory T cells that express the FOXP3+ transcription factor (FoxP3+ Tregs); the most relevant was PTX3. Until recently, it was known that for solid tumors, in particular for pancreatic carcinoma, elevated gene expression of PTX3 corresponds to poor prognosis [69]. Recent findings have established a significant correlation between elevated gene expression of PTX3 and poor prognosis in hematological malignancies. In cases of non-GCB DLBCL, elevated levels of CD16, CD163, PTX3, and IL-10 were observed [67].

### 3.4. CREBBP/EP 300 Mutations

The epigenetic mutations are significant in DLBCL progression, and their biological relevance needs further observation. In newly diagnosed cases of diffuse large B-cell lymphoma (DLBCL), several genes have been identified that are associated with immune cells and clinical outcomes. These genes include those involved in histone methylation (KMT2D, KMT2C, EZH2), histone acetylation (CREBB, EP300), DNA methylation (TET2), and chromatin remodeling (ARID1A). The most often involved were somatic mutations in KMT2D [70], followed by mutations in ARID1A, TET2 [71], CREBBP, EP300 [72], KMT2C, and EZH2 [73]. Poor clinical outcomes were observed in cases with CREPP/EP300 mutations, and the ratio of lymphocyte/monocyte in the peripheral blood was low [73]. In the murine B-cell neoplasms example, the cases with chromatin-modifying gene CREBBP or EP300 mutations that had a decreased H3K27 acetylation led to higher M2 polarization and a faster tumor progression when compared to CREBBP/EP300 wild-type, regulated via the FBXW7-NOTCH-CCL2/CSF1 axis [73]. Mutations in the CREBBP/EP300 gene are commonly observed in hematological malignancies and can arise through a range of mechanisms. These mutations are associated with unfavorable patient prognoses, which include reduced overall survival (OS), progression-free survival (PFS), and event-free survival (EFS) [74]. Mutations in the CREBBP/EP300 gene are also often identified in other types of lymphomas besides DLBCL, including FL [75,76], in situ follicular neoplasia [77], peripheral T-cell lymphoma (PTCL) [78], angioimmunoblastic T-cell lymphoma (AITL), and plasmablastic lymphoma [74]. CREBBP/EP300 mutations are a key pathogenetic mechanism in B-cell non-Hodgkin lymphoma (B-NHL) and have important implications for drugs targeting acetylation and deacetylation pathways [74]. Histone deacetylase inhibitors (HDACis) have shown promising roles in the treatment of patients with follicular lymphoma (FL), particularly those with nonfunctional CREBBP [79]. These findings indicate that HDACis could be a beneficial option for patients with B-NHL, as they may help restore normal acetylation levels and enhance tumor immune surveillance. However, it is crucial to assess their efficacy and target specificity in clinical contexts [74]. By focusing on specific gene mutations, a Phase 1 study evaluated the safety profile and pharmacokinetics of CCS1477 inobronib, addressing the underlying molecular abnormalities in certain hematologic malignancies [80]. In a Phase 2 study examining the efficacy of mocetinostat, an oral HDACi, researchers investigated its effects on patients with relapsed or refractory diffuse large B-cell lymphoma (DLBCL) or follicular lymphoma (FL) who have mutations in the CREBBP/EP300 genes. The study was registered at www.clinicaltrials.gov (NCT02282358). The aim was to determine whether mocetinostat could slow the progression of cancer. Treatment continued until disease progression occurred, intolerable side effects developed, or the patient passed away. The clinical benefits suggest the potential efficacy of mocetinostat. Additionally, the manageable safety profile indicates that further investigation, especially in combination with other therapeutic agents, is warranted [81]. Another Phase 2 study evaluated the efficacy of combining tucidinostat (formerly known as chidamide), an oral histone deacetylase inhibitor (HDACi), with R-CHOP chemotherapy in elderly patients recently diagnosed with diffuse large B-cell lymphoma (DLBCL). The results are available at www.clinicaltrials.gov (NCT02753647). The findings indicate that this combination therapy is both effective and well tolerated in the patient cohort studied. Furthermore, the research highlights the potential implications of CREBBP and EP300 mutations on treatment outcomes [82]. The integration of an AURKA inhibitor in conjunction with chidamide constitutes a novel and potentially effective therapeutic approach for the treatment of relapsed or refractory diffuse large B-cell lymphoma (DLBCL) [83].

### 3.5. CCL2/CCR2 Axis in Double-Expressor DLBCL

The pathogenic mechanisms underlying double-expressor diffuse large B-cell lymphoma (DE-DLBCL), specifically related to the myelocytomatosis oncogene (MYC) and B-cell lymphoma 2 (BCL2), are not yet fully understood. Additionally, the roles of MYC and BCL2 in influencing lymphoma progression and developing resistance to conventional treatments warrant further investigation [84,85]. DE-DLBCL stands up to 30% of DLBCL cases and is correlated with the non-GCB subtype, a higher Ki-67% index, and elderly cases, being an independent poor outcome marker [86]. The mechanisms driving the aggressiveness of DE-DLBCL remain unclear, and treatment approaches for cases with and without DE-DLBCL are largely similar [87]. For further investigation related to this matter, transcriptome analysis was conducted, and a higher amount of messenger RNA of C-C motif chemokine ligand 2 (CCL2) and C-C chemokine receptor type 2 (CCR2) was seen in cases of DE-DLBCL [84]. These are important molecules involved in monocyte enlistment and M2 macrophage polarization [88,89]. Significantly increased levels of M2 macrophages and lower T-cell infiltration for DE-DLBCL cases were observed [84]. The prognostic involvement of CCL2/CCR2 expression corresponding to the cell of origin or MYC and BCL2 state was surveyed. Separately from the cell of origin, in DE-DLBCL and non-DE-DLBCL cases, high CCL2/CCR2 conditions were linked with inferior outcomes [47]. Starting from the fact that an expanded expression of CCL2 was observed in tumor-assaulting macrophages, not in DLBCL cells, it was presumed that DE-DLBCL cells could produce CCL2 and influence CCR2-expressing macrophages [84]. It was revealed that MYC and BCL2 were associated with raised CCL2 expression by upregulating nuclear factor-kB in tumor cells and promoting M2 polarization [84]. It was demonstrated that the CCL2/CCR2 axis is associated with tumor aggressiveness in DE-DLBCL by increasing M2 polarization rates, making it a potential therapeutic target. Ongoing studies analyze the BCL-2 inhibitor, venetoclax, as a first-line treatment option alongside R-CHOP, particularly for BCL2+ DLBCLs and DE cases [32]. Immunotherapy with antigen receptor T-cell therapy and a bispecific T-cell engager (bispecific Ab) has favorable results in DLBCL cases [90]. The CCL2-CCR2 axis is an important pathway in chemokine signaling. In addition to its role in diffuse large B-cell lymphoma (DLBCL), it is associated with various other conditions, such as psoriasis, rheumatoid arthritis, atherosclerosis, and cardiovascular diseases. This axis is also considered a significant biomarker for several types of cancer, including malignant melanoma, ovarian cancer, colorectal cancer, bladder cancer, kidney cancer, lung cancer, and breast cancer [91].

### 3.6. Neuron-Specific Enolase

A previous study investigated the correlation between neuron-specific enolase (NSE) or enolase 2 (ENO2) and macrophage polarization in DLBCL [92]. NSE is an isoenzyme of enolase, an enzyme with glycolytic properties whose appearance occurs towards the end of neural formation, used as a mark of neuronal development [93]. Elevated ENO2 levels are considered a negative predictive marker in tumors of neuroendocrine lineage, as well as in those arising from epithelial tissues like breast cancer, non-small cell lung cancer, and lymphoid tissues like DLBCL [93]. It has been found that lymphoma patients with higher levels of ENO2 tend to have a worse survival prognosis, particularly those with non-germinal center DLBCL [92]. Previous studies illustrated that ENO2 promotes macrophage polarization, leading to an M2-like phenotype, and a recent investigation studied how DLBCL-derived exosomal ENO2 modulates macrophage polarization, leading to DLBCL progression [94]. Exosomes are extracellular vesicles that transfer a wide range of proteins, lipids, RNAs, and DNAs [95], also involved in regulating immune cells in the TME [96,97]. It was determined that high ENO2 levels are linked to an elevated M2/M1 ratio in the TME and a poor OS in DLBCL. After relapse, DLBCL cases were shown to have elevated ENO2 levels compared to those at diagnosis. A higher level was determined for stage III/IV DLBCL patients. DLCBL exosomes were fixed by THP-1 macrophages and, via ENO2, promoted macrophage polarization to the M2 subtype, leading to proliferation, migration, and invasion of DLBCL [94]. The adjustment of macrophage polarization by tumor-derived ENO2 is modulated by glycolysis [98].

### 3.7. Recombinant Hirudin and PAR-1

Given the strong association between macrophage polarization and cancer proliferation, recent research has concentrated on recombinant hirudin (rH) and its regulatory role in macrophage polarization to discover new targeted drugs [99]. A connection between cancer progression and blood clots was observed, as thrombin promotes angiogenesis and supports the proliferation of cancer cells [99]. It was established that thrombin is linked to protease-activated-receptor-1 (PAR-1) to promote tumor progression [99]. It is important to find new curative options for DLBCL individuals resistant to standard therapies [99]. The OS rate for DLBCL cases treated with rituximab is 65% in five years [100]. Abnormalities in the tumor microenvironment significantly influence tumor development [21]. Due to changes in their microenvironment, macrophages can exhibit different activation forms [101]. The imbalance between M1 and M2 types is linked to a range of serious health conditions, including cancer [102], cardiovascular diseases [103], liver disorders [104], and neurological deterioration [105]. Many studies have reported the M2 subtype as tumor-resistant [104], connected to a bad prognosis [106]. The influence of different pharmaceutical agents on thrombin was evaluated during a clinical trial focused on lymphoma, but there is insufficient data about PAR-1 in DLBCL [107]. Research on thrombin and PAR-1 in diffuse large B-cell lymphoma (DLBCL) is limited. While drugs like Vorapaxar and Atopaxar hydrobromide target PAR-1 [107], additional studies are necessary to confirm the efficacy of PAR-1 inhibitors in cancer treatment [99]. Beginning with the understanding that hirudin has anticoagulant properties and that recombinant hirudin (rH) functions as an inhibitor of coagulation, recent considerations suggest that rH may represent a significant therapeutic option. Its use may help reduce proliferation rates in various cancers and potentially halt metastasis [108]. Recent studies established that rH can regulate macrophage polarization, promoting the M1 subtype and suppressing M2 polarization, suggesting the potential curative role of hirudin in DLBCL [99]. Also, PAR-1, an important molecular target in malignancies, is elevated in DLBCL cases. Knockdown of PAR-1 induces the conversion of M2 macrophages to the M1 subtype, while its overexpression can promote metastasis [109]. Therapeutic approaches using hirudin, particularly in combination with paclitaxel, may reduce inflammation in cardiovascular diseases [110]. The potential therapeutic combination of rituximab and hirudin for enhancing treatment outcomes in non-Hodgkin lymphoma (NHL) cases warrants further investigation [99]. A study involving 32 clinical samples from DLBCL found that rH inhibited the M2-type polarization and modulated PAR-1, influencing tumor progression, but there are no ongoing clinical trials specifically for lymphoma treatment. However, recombinant hirudin has been investigated in other types of cancer. A study explored the effects of direct thrombin inhibitors, such as rH, in non-small cell lung cancer (NSCLC), showing that they suppressed tumor progression, dissemination, and metastasis [111].

### 3.8. Expression of miR-155

It was proposed that the deletion of miR-155 in macrophages and dendritic cells is associated with a precancerous effect, leading to tumor expansion [43]. MicroRNAs (miRNAs) are small, single-stranded RNA molecules, typically 17 to 25 nucleotides long, that play a crucial role in regulating gene expression [112]. They can suppress protein production by degrading the mRNA transcript or inhibiting translation. This regulation occurs through the complementary binding of miRNAs to mRNA, leading to either degradation or translation inhibition [113]. MiR-155 has a critical role in promoting M1 macrophage activation, selecting inflammatory cells, and enhancing the antitumor response during carcinogenesis [114]. Higher levels of miR-155 were associated with a decreased rate of CD163 and CD68 in patients with EBV-negative DLBCL [115]. A potential new therapeutic approach for DLBCL EBV-positive cases involves targeting pro-tumoral macrophages and regulating epigenetics [115]. MicroRNAs play a significant role in developing B-cell processes and B-cell lymphoma [116]. Some were related to an oncogenic role alongside miR-155, like the miR-17-92 cluster, miR-21, miRNA, and miR-217 [117]. MiR-17-92 is believed to be a powerful driver of cancer [117]. It was found that miR-217 behaves like an oncogene in GC B cells, showing an increased expression in these cells [117]. Another observation related to miR-21 was made, which seems to be involved in oncogenesis. It appears to have an upregulated expression in most tumors analyzed, like breast cancer, colon cancer, and glioblastoma [73,118,119]. In contrast to the miRNAs mentioned above, the following act as tumor suppressor genes: miR-181a, miR-34a, miR-146a, Cluster 15a/16-1 miRNAs, and miR-28. MiR-181a downregulates oncogenic signaling, decreases proliferation, and slows down the rate of tumor expansion [120]. Developing new therapeutic approaches for B-cell lymphomas could be possible if the expression of miRNAs was controlled.

### 3.9. PD-L1

Expressions of programmed death-ligand 1 (PD-L1) and its receptor, programmed cell death-1 (PD-1), are observed across a variety of cell types, like tumor cells (TCs) or antigen-presenting cells, T cells, reducing the activation of immune cells (ICs) [121]. Elevated levels of PD-L1 expression and soluble PD-L1 have been linked to poorer and improved clinical outcomes in patients with de novo DLBCL [122]. This variation may result from differences in patient populations, the use of various PD-L1 reagents, or the complex biology of PD-L1, which is associated with the heterogeneous nature of this disease [123]. Limited data support the use of single-agent therapy targeting PD-1/PD-L1 in DLBCL. There is a need for a better understanding of this biological complex, as well as more clinical studies. In newly diagnosed cases of DLBCL treated with immunochemotherapy, the expression of PD-L1 in tumor B cells has been linked to poor clinical outcomes [121]. A study grounded in the hypothesis that PD-L1 expression correlates with activated tumor-infiltrating macrophages and may not be associated with poor prognosis in patients with de novo DLBCL undergoing chemoimmunotherapy is in contrast with findings in solid tumors. Utilizing samples from two major phase 3 trials, the investigation seeks to enhance our understanding of the biological and clinical significance of PD-L1 expression in de novo DLBCL. Notably, the results demonstrate that PD-L1 is expressed in up to 95% of myeloid cells among patients with de novo DLBCL, while expression in tumor cells is limited to approximately 10% [121]. The primary source of PD-L1 expression was the tumor-infiltrating macrophages, and the signaling pathway involved STAT3 [121]. Starting from the premise that PD-L1 expression may indicate a diverse array of activated tumor-infiltrating macrophages relevant to the anti-CD20 response, a correlation was established between PD-L1 and macrophage gene expression. This association has been identified as a potential marker of improved prognosis in select cases. Research has indicated that elevated expression of a macrophage signature is associated with improved survival outcomes in de novo DLBCL patients undergoing immunochemotherapy. Furthermore, this increased expression correlates with extended progression-free survival (PFS), particularly within the ABC subtype, which is known to have a poorer prognosis in clinical settings. Determining the independent contribution of each macrophage subtype was challenging; only the M0 (resting) subset was linked to PFS. Additional research should focus on the effects of PD-L1-expressing myeloid cell subsets on clinical outcomes and phagocytic activity, with implications for therapies targeting anti-CD20, anti-PD-1/PD-L1, and other myeloid-specific treatments such as anti-CSF1R [121]. A Phase 2 open-label study was conducted on 46 previously untreated patients with high-risk DLBCL to evaluate durvalumab, a high-affinity human IgG1 monoclonal antibody that selectively blocks PD-L1 binding to PD-1 and CD80. Data are registered at https://clinicaltrials.gov (NCT03003520). The patients were divided into two arms: the first arm received durvalumab in combination with R-CHOP chemotherapy, while the second arm was treated with durvalumab, lenalidomide, and R-CHOP. Treatment continued until disease progression or unacceptable toxicity occurred. According to the latest available data, the study showed that higher PD-L1 expression on tumor cells is correlated with better outcomes.

## 4. Discussion

One of the key challenges in current clinical trials targeting TAMs in DLBCL is the heterogeneity of TAM phenotypes and their functions. This heterogeneity and plasticity complicate the development of therapies that can selectively modulate TAMs. The exact mechanisms of TAMs in DLBCL are still being unraveled, and their interactions with other immune cells, such as dendritic cells, are complex and poorly understood. TAM-targeting agents are used in combination with other treatments, like chemoinmunotherapy or checkpoint inhibitors, but optimal combinations and treatment strategies are not yet well-defined.

The study of galectin family members as a novel therapeutic target in lymphoma therapy can lead to overcoming chemotherapy resistance using targeted therapies. However, there are some limitations due to scarce clinical data and challenges in drug development.

High PTX3 expression is linked to poor prognosis in DLBCL, having significant meaning regarding risk stratification. The study was conducted on a large cohort, enhancing the reliability of the results. The findings highlight PTX3 as a potential treatment in lymphoma. It is essential to recognize that the study only demonstrates a correlation, and further research is needed.

Based on a large cohort, using well-defined experimental models and comprehensive genetic analyses, the first report on the impact of CREBBP/EP300 mutation on TAMs was made. This can lead to novel therapeutic targets in treating DLBCL. Since cell lines and animal models were used, the impact of these mutations on TAM polarization and tumor progression needs further investigation for clinical relevance. Data from preclinical and clinical studies on epigenetic therapies in lymphoma indicate that HDAC inhibitors, such as chidamide, can enhance the effect of doxorubicin. This combination specifically sensitizes double BCL2/MYC-expressing DLBCLs to conventional chemotherapy. Further clinical trials with a larger, well-randomized population are essential to recommend chidamide for patients with B-NHL.

The identification of the CCL2/CCR2 axis as a factor related to poor prognosis in DLBCL patients adds clinical relevance and may influence future therapeutic strategies.

The study regarding ENO2 explores the role of exosomal ENO2 in macrophage polarization, providing new insights into the interactions between DLBCL cells and the tumor microenvironment. The use of bioinformatics to correlate ENO2 expression with DLBCL progression and macrophage polarization ratios strengthens the validity of the findings. The combination of in vitro and in vivo experiments enables a thorough investigation of the mechanisms at play, thereby enhancing the reliability of the results. The identification of exosomal ENO2 as a therapeutic target and prognostic biomarker underscores its significance in clinical settings, potentially leading to enhanced treatment strategies for DLBCL. While the study shows promising laboratory results, further validation in clinical cohorts is necessary to confirm the utility of exosomal ENO2 as a biomarker or therapeutic target for DLBCL.

The study examining the effects of rH and PAR-1 in DLBCL uncovers a novel mechanism for rH influence on macrophage polarization. This finding enhances our understanding of the immune environment in DLBCL and highlights the regulation of PAR-1 as a potential therapeutic pathway. By exploring the interaction between rH and macrophages, this research proposes a targeted approach that may improve treatment strategies and patient outcomes in DLBCL and other immune-related diseases. The findings are limited by the lack of clinical data and the interactions between rH and other drugs used in DLBCL.

Recent data indicate that PD-L1 did not effectively identify high-risk patients in de novo DLBCL and may be linked to better prognosis in some cases. The explorations of how TAMs influence treatment outcomes highlight potential therapeutic targets and strategies, while the complexity of macrophage polarization can complicate interpretation of the results.

## 5. Conclusions

Non-Hodgkin lymphoma is the most prevalent type of hematological cancer globally. The eradication of aggressive forms of the disease is highly desirable. Up to 40% of cases fail with standard treatments, highlighting the need for new therapeutic methods. Macrophage polarization significantly impacts the tumor microenvironment of DLBCL, with implications for both disease progression and therapeutic efficacy. This effect arises from the capability of macrophages to dampen the immune response to cancer cells and to inhibit apoptosis. The shift between pro-inflammatory M1 and immunosuppressive M2 macrophages may serve as a valuable biomarker for disease progression and treatment efficiency. High levels of galectin-3 are associated with poor prognosis, as they play a role in cancer cell adhesion to the extracellular matrix and exhibit anti-apoptotic actions. M2 macrophage polarization markers, including CD68 and CD163, are associated with poor prognosis. PTX3 is a newly identified marker linked to negative outcomes in hematological cancers. Higher levels were observed in non-GCB DLBCL. Epigenetic mutations play a significant role in DLBCL, and cases with wild-type CREBBP/EP300 exhibited faster disease progression. However, their significance requires further study. M2 polarization, enhanced by the CCL2/CCR2 axis, correlates with cancer aggressiveness in DE-DLBCL, presenting a potential therapeutic target. Exosomal ENO2 derived from DLBCL actively modulates macrophage polarization, driving the progression of DLBCL. Harnessing the ability to control miRNA expression could pave the way for groundbreaking therapeutic strategies in the fight against B-cell lymphomas. Targeting macrophage polarization, either alone or with existing therapies, holds promise for improving outcomes in DLBCL. Further research into the molecular mechanisms of macrophage interactions is essential for developing more effective and personalized treatments.
